# An Optimized Procedure for the Site-Directed Labeling of NGF and proNGF for Imaging Purposes

**DOI:** 10.3389/fmolb.2017.00004

**Published:** 2017-02-02

**Authors:** Pierluigi Di Matteo, Mariantonietta Calvello, Stefano Luin, Laura Marchetti, Antonino Cattaneo

**Affiliations:** ^1^BioSNS Laboratory, Scuola Normale Superiore and Istituto di Neuroscienze – CNRPisa, Italy; ^2^NEST Laboratory, Scuola Normale Superiore and Istituto Nanoscienze – CNRPisa, Italy; ^3^Center for Nanotechnology Innovation@NEST, Istituto Italiano di TecnologiaPisa, Italy

**Keywords:** fluorescent NGF, fluorescent proNGF, neurotrophins labeling, neurotrophins purification, signaling endosome

## Abstract

Neurotrophins are growth factors of fundamental importance for the development, survival and maintenance of different neuronal and non-neuronal populations. Over the years, the use of labeled neurotrophins has helped in the study of their biological functions, leading to a better understanding of the processes that regulate their transport, traffic, and signaling. However, the diverse and heterogeneous neurotrophin labeling strategies adopted so far have often led to poorly reproducible protocols and sometimes conflicting conclusions. Here we present a robust, reliable, and fast method to obtain homogeneous preparations of fluorescent proNGF and NGF with 1:1 labeling stoichiometry. This strategy is well suited for several applications, ranging from advanced imaging techniques such as *single particle tracking*, to analyses that require large amounts of neurotrophins such as *in vivo* monitoring of protein biodistribution. As a proof of the quality of the labeled NGF and proNGF preparations, we provide a quantitative analysis of their colocalization with proteins involved in the *signaling endosome* function and sorting. This new analysis allowed demonstrating that proNGF localizes at a sub-population of endosomes not completely overlapped to the one hosting NGF.

## Introduction

Neurotrophins (NTs) exert a multitude of biological functions both in the central and peripheral nervous system, related to neuronal development, adult physiology, and neurodegenerative processes.

The use of labeled neurotrophins has represented, over the years, a valuable experimental strategy to investigate NT functions. Several methods to chemically label NTs have been presented in the literature, spanning from radioiodinated NGF (Frazier et al., 1974; Max et al., 1978) to chemically-labeled NTs (Rosenberg et al., 1986; Bronfman et al., 2003; Shibata et al., 2006; Cui et al., 2007; Echarte et al., 2007; Nomura et al., 2011). In all these methods, the chemical labeling is not site-directed at defined residues of the NT molecule and the labeling stoichiometry is poorly controlled. For intracellular imaging, NT-GFP fusions have been used (Mowla et al., 1999; Adachi et al., 2005), but the large size of the GFP moiety is not ideal. All these neurotrophin labeling methods often caused poorly reproducible results and sometimes inconsistent conclusions. Thus, there is a great need for a new generation of site-directed NT labeling methods exploitable for a variety of applications.

Building on work by Sung et al. (2011), our group has recently developed a new site-directed and stoichiometry-controlled labeling of NGF and proNGF (Marchetti et al., 2014; De Nadai et al., 2016). Accordingly, the coding sequence of human proNGF can be modified by the C-terminal insertion of the YBBR tag coding sequence (De Nadai et al., 2016). This 11-amino-acid long peptide (DSLEFIASKLA) was identified from a series of truncated proteins derived from the *ybbR* ORF gene of *Bacillus subtilis* and shown to be an efficient substrate for the enzymatic activity of the Sfp synthase enzyme (Yin et al., 2005). Once translated, this tag is specifically recognized by this enzyme, which covalently conjugates the phosphopantetheinyl arm of a Coenzyme A (CoA) substrate to a specific serine in the YBBR sequence (Yin et al., 2005). Thus, the use of fluorescent CoA derivatives (e.g., CoA-Alexa647) allows to produce fluoNGF and fluoproNGF (De Nadai et al., 2016). This labeling strategy has several advantages: (i) it allows for the purification of fully functional labeled precursor and mature neurotrophins; (ii) the controlled, site-specific fluorolabeling allows the production of homogeneously labeled NTs, suitable for quantitative analysis; (iii) it is versatile, allowing to covalently conjugate different derivatives of CoA, depending on the purpose.

This labeling strategy has allowed comparing, for the first time, the transport properties of proNGF and NGF at the level of single vesicles in living cells (De Nadai et al., 2016). Due to the potential of this new NT labeling method, there is a big interest to optimize the procedure and to facilitate the production of fully validated labeled NTs. In this work we present an optimized robust and reproducible protocol for the purification of fluorescent NGF and proNGF (hereafter referred as fluoNGF and fluoproNGF) with higher yield, specific activity, and purity degree. Obtained fluoproNGF and fluoNGF were used in an internalization assay in differentiated PC12 cells and probed for their colocalization with proteins previously identified to regulate the trafficking and sorting of NGF *signaling endosomes*, such as Rab11, Rab7, Rab5, and EEA1 (Liu et al., 2007; Sorkin and von Zastrow, 2009; Wu et al., 2009). Data show that the divergent signaling promoted by these two NT forms relies also on distinct internalization routes undertaken by the two of them.

## Materials and methods

### Protein expression

All chemical reagents were purchased from Sigma-Aldrich. The plasmid containing the sequence for the recombinant expression of the human proNGF-YBBR has been previously obtained by the insertion of the YBBR coding sequence downstream the sequence encoding for proNGF (De Nadai et al., 2016). *Escherichia coli* BL21 strain was transformed with 100 ng of plasmid. The cells were plated onto a petri dish containing Luria Bertani (LB) medium supplemented with agar and ampicillin, and grown overnight at 37°C.

The day after, one colony was picked and inoculated in 20 ml of LB medium supplemented with ampicillin and grown overnight at 37°C with shaking at 250 rpm. Then, 18 ml of this culture were inoculated in 1 L of LB medium supplemented with ampicillin, splitted into five flasks each containing 200 ml and allowed to grow until an OD_600_ of about 1 was reached, before inducing protein expression with 1 mM of IPTG (Isopropil-β-D-1-tiogalattopiranoside) for 5 h at 37°C.

### Purification of inclusion bodies

Expressing bacteria were centrifuged at 6000 rpm, 4°C for 10 min. The obtained pellets were resuspended in 20 ml of lysis buffer (10 mM Tris-HCl pH 8; 1 mM EDTA pH 8, and 1 mg/ml lysozyme) and allowed to stand for 1 h at room temperature. This solution was sonicated three times (45″ ON, 60″ OFF at 4°C) and finally DNAse (50 μg/ml) supplemented by MgCl_2_ (5 mM) was added. After 30 min, 10 ml of Triton buffer was added, consisting of 60 mM EDTA, 1.5 M NaCl, and 6% v/v TRITON X-100 (Sigma-Aldrich).

After 30 min of gentle shaking, the solution was transferred into two glass centrifuge tube (Corex) and centrifuged at 13,000 rpm, 4°C for 30 min. The supernatant was discarded, and the pellet was resuspended in 20 ml of buffer containing: 10 mM Tris-HCl pH 8 and 1 mM EDTA. At this point 10 ml of Triton buffer was added to the solution, incubated for 30 min in gentle shaking, finally followed by centrifugation at 13,000 rpm, 4°C for 30 min. The pellet was washed three times using 25 ml of buffer containing: 50 mM Tris-HCl pH 7.5 and 1 mM EDTA, adopting for each step the previously mentioned centrifugation settings.

### Protein refolding

To allow the proper denaturation of the neurotrophin, the obtained pellet was solubilized in 5 ml of Guanidinium buffer (6 M Guanidinium chloride; 100 mM TrisHCl pH 8; 1mM EDTA; 100 mM Dithiothreitol). Then hydrochloric acid was added until the solution reached pH 3.5. At this point the solution was centrifuged at 13,000 rpm, 4°C for 30 min. The supernatant, containing the protein of interest, was dialyzed in 300 ml of 6 M Guanidinium chloride pH 3.5 for 36 h, changing the buffer every 12 h.

After dialysis, the protein concentration was measured and 5 mg of neurotrophin was added every hour to 100 ml of refolding buffer (1 M Arginine pH 9.3; 100 mM Tris-HCl pH 9.3; 5 mM EDTA pH 8; 1 mM Glutathione disulphide and 5 mM reduced Glutathione) at 4°C. After completing the refolding step, the buffer was exchanged by dialysis with one containing 50 mM sodium phosphate buffer pH 7 and 1 mM EDTA. After overnight dialysis, the solution was centrifuged at 7500 rpm for 5 min and then filtered (0.22 micron pore size).

### Neurotrophins purification

To obtain a pure solution of proNGF-YBBR, the refolded neurotrophin was purified by ion-exchange FPLC, using HiLoad™ 16/10 SP Sepharose™ High Performance column (GE Healthcare Life Sciences) and the following mobile phase buffer: 50 mM sodium phosphate buffer pH 7; 1 mM EDTA with 0 M or 1 M NaCl respectively in buffer A or buffer B. A linear gradient from 0 to 100% of buffer B in eight column volumes was applied to elute the protein, at 1 ml/min of flow rate. Fractions of 3 ml were collected. A small aliquot (20 μl) of each fraction was assayed by SDS-PAGE. Fractions containing the protein were pooled obtaining about 18 mg of proNGF-YBBR at a final concentration of 1 mg/ml. Four milligrams of protein were dialyzed overnight in a buffer containing 50 mM sodium phosphate buffer pH 7 and 150 mM NaCl (storage buffer), and finally aliquoted. To ascertain the integrity of the purified neurotrophin, 100 μg of protein was analyzed by mass spectrometry (MS). MS analysis was performed by an external facility (Toscana Life Sciences Foundation).

Mature neurotrophin was obtained by controlled proteolysis of the purified proNGF-YBBR, using trypsin protease (1 μg of enzyme for 300 μg of neurotrophin, 16 h at 4°C). 14 ml of solution containing proNGF-YBBR was digested and then diluted in 100 ml of FPLC buffer A. Another step of FPLC chromatography was used for the purification of mature NGF-YBBR; three peaks were found in the chromatogram. Twenty microliters of each collected fraction were analyzed by SDS-PAGE in this case as well. Small amounts (100 μg each) from three different fractions (32, 45, 58 as in **Figure 2**) were analyzed by MS. The obtained fractions of NGF-YBBR were pooled in this way: from fraction 32 to 39 (1° peak), from 42 to 50 (2° peak), and from 52 to 62 (3° peak). The three samples were dialyzed overnight in the storage buffer and separately aliquoted. The stability of the YBBR proNGF and NGF proteins upon storage at −80°C and at a concentration of 1 mg/ml was found not to be qualitatively different from that of their wild type counterparts.

### fluoproNGF and fluoNGF production

CoA-Alexa647 substrate was synthesized starting from 200 nmol of Alexa647 maleimide (Thermo Fisher Scientific) and 260 nmol of CoA trilithium salt (Sigma Aldrich) dissolved in 20 mM HEPES pH 7.2 up to a final volume of 240 μl. The solution was incubated for 4 h at 350 rpm and 37°C, and it was then purified by Reverse Phase HPLC using Phenomenex Fusion-RP phase column and 10 mM ammonium formate/acetonitrile as mobile phase. The purified adduct was quantified, lyophilized in ≈50 nmol aliquots, resuspended in H_2_O and stored at −20°C. Sfp Synthase was either produced in *E. coli* as a His-tagged protein as previously described (Yin et al., 2006), or purchased at New England Biolabs (NEB).

For the fluorolabeling reaction of proNGF-YBBR, 85 μg of neurotrophin were incubated for 30 min at 37°C and 350 rpm in a reaction mix composed by: 76 μM CoA-Alexa647, 4.8 μM Sfp Synthase (NEB), 38 mM MgCl_2_, in phosphate buffer (#D8662 Sigma Aldrich) up to 260 μl final volume. For the fluorolabeling of NGF-YBBR, 90 μg of neurotrophin were incubated in the same conditions of the proNGF-YBBR in buffer containing: 73 μM CoA-Alexa647, 17 μM Sfp Synthase, 36 mM MgCl_2_, in phosphate buffer up to 270 μl final volume.

We found that performing the labeling reaction in the same tubes used to store the aliquots of the neurotrophin is critical in order to avoid massive loss of protein. After adding phosphate buffer, the reaction tube was spun at 7000 rpm for 1 min at 4°C. Then the remaining reagents were added. After the reaction was complete, the reaction tube was immediately placed on ice.

In order to separate the fluorescent neurotrophins from the non-reacted ones, the reaction mix was processed by cation-exchange HPLC, keeping the temperature of the sample at 4°C after the chromatographic run, using Propac SCX-20 column (Dionex, Thermo Fisher Scientific) and 100 mM HNa_2_PO_4_ mobile phase with 0 M to 1 M NaCl gradient. The fluorescent proNGF and NGF proteins were stored at 4°C at a concentration of about 4 and 14 μg/ml, respectively, and were found to be stable, as fluorescent proteins, for 15–20 days.

### Immunocytochemistry

PC12 cells (ATCC, CRL-1721) were maintained at 37°C, 5% CO_2_ in RPMI 1640 GlutaMAX medium (Thermo Fisher Scientific) supplemented with 10% horse serum, 5% fetal bovine serum, and 1% penicillin/streptomycin from Gibco (complete medium). Cells were first primed for differentiation, i.e., maintained at 50–70% confluency in complete medium supplemented with 15 ng/ml of wt recombinant human NGF for 6 days, with 70% of the medium renewed every 2 days.

After 6 days of priming, cells were harvested and the medium completely replaced with RPMI 1640 GlutaMAX supplemented with 1% horse serum, 0.5% fetal bovine serum, 1% penicillin/streptomycin, and 50 ng/ml of wt recombinant human NGF (differentiation medium). Cells were plated on Poly-L-lysine coated 8 well Glass micro-Slide (Ibidi) microscope slides, at 20,000 cells/cm^2^ density. All medium was renewed every 48 h. Four days of differentiation are necessary to complete the process.

At the end of the differentiation process, medium was replaced with sterile phosphate buffer with Calcium and Magnesium (#D8662 Sigma Aldrich) in order to wash out residual NGF traces. After 1 h, the medium was replaced with differentiation medium containing fluoproNGF (200 ng/ml) or fluoNGF (100 ng/ml). After 3 h of incubation with each fluoNT, cells were fixed using 4% paraformaldehyde and 4% sucrose in phosphate buffer for 15 min. Cells were then permeabilized in phosphate buffer containing 8% of Bovine Serum Albumin (Sigma Aldrich) and 0.1% Saponin (Sigma Aldrich) for 7 min. After five washes in phosphate buffer supplemented with 8% BSA, cells were blocked using the same solution for 1 h. The primary antibodies (Anti-Rab11 Cell Signaling #5589, 1:200; Anti-Rab7 Cell Signaling #9367, 1:200; Anti-Rab5 Cell Signaling #3547, 1:300; Anti-EEA1 Cell Signaling #3288, 1:200) were administered for 16 h at 4°C, diluted in blocking solution. The secondary antibody (Anti-Rabbit conjugated to Alexa488 fluorophore, 1:500, Life Technologies) was administered for 2 h at room temperature. The cells were washed three times with phosphate buffer and once with Milli-Q water (Millipore), dried and maintained in mounting medium (Fluoroshield, Sigma).

### Microscopy measurements

Images were captured using a confocal laser scanning microscope (Leica TCS SP5 SMD), equipped with 63X/1.2 NA objective. 0.7 airy unit pinhole was adopted. Fluorescence signals were acquired using Ar 488 and 633 solid state lasers sequentially. PMT detection bandwidths were 503–570 nm for Alexa488 and 645–767 nm for Alexa647. The images were acquired by maintaining a pixel size of ~90 nm and an image dimension of 512 × 512 pixels. About 20 cells were analyzed for each experimental condition.

### Image analysis

Colocalization analysis was performed by custom made Matlab scripts. Images were subjected to pixelwise adaptive Wiener filtering for noise removal (Matlab function wiener2; estimation of mean, standard deviation, and noise power in a 15-by-15 pixels window), local background subtraction (Gaussian blurring with a 15-pixels- wide Gaussian kernel), and Gaussian smoothing (3 pixels). Colocalization was quantified using a Manders colocalization coefficient (Dunn et al., 2011), with thresholds set at 10% of the maximum in the single image. The script returns the percentage of NT's fluorescence signal that colocalizes with the signal of the considered protein partner, among the total neurotrophin signal.

### Statistical analysis

Statistical analysis of data was performed using OriginLab software. The differences in the colocalization percentage between NGF and proNGF with endosomal partners were analyzed by two-tailed Student's *t*-test. For all the analyses α = 0.05 was adopted as a level of significance.

## Results

### proNGF-YBBR and NGF-YBBR expression and purification

**Figure 1:**
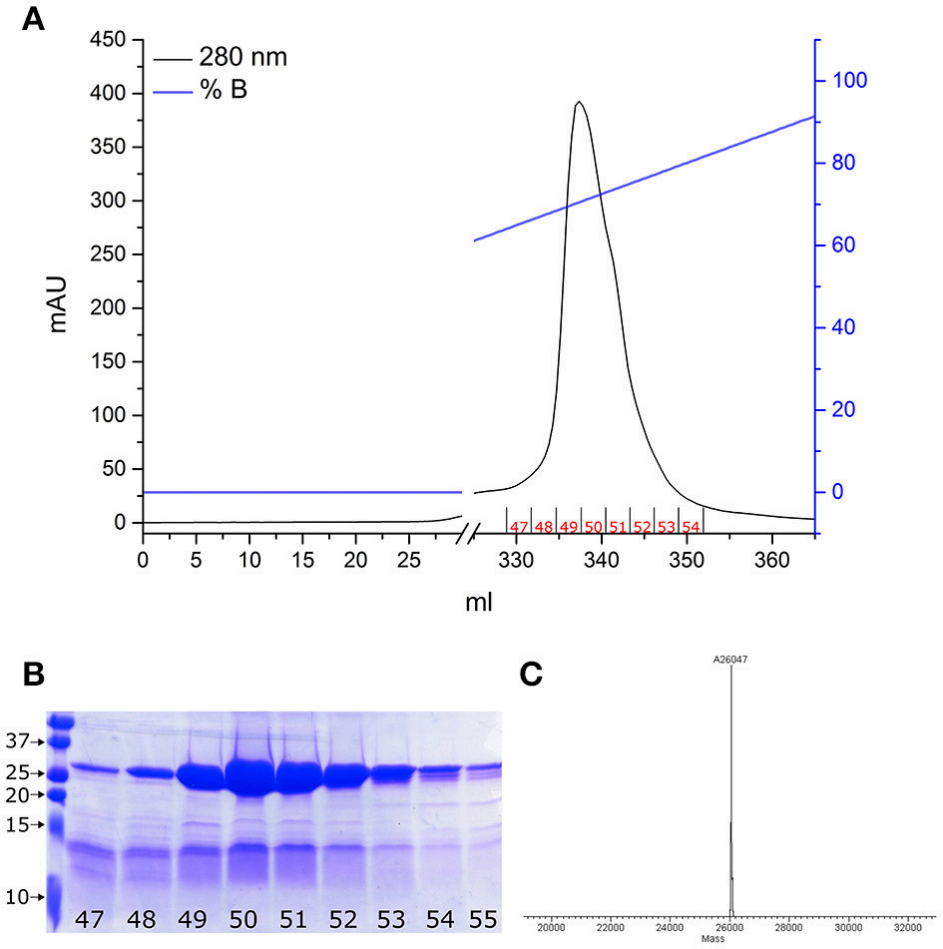
**proNGF-YBBR purification. (A)** Chromatographic profile of FPLC analysis. Black line and blue line represents respectively the absorbance at 280 nm and the percentage of buffer B on the total flowing buffer. The numbers identifying the collected fractions are shown in red. **(B)** SDS-PAGE analysis of the collected fractions corresponding to the proNGF-YBBR peak. On the left side of the gel, molecular standard weights (kDa) are shown. Numbers of each lane correspond to the fraction analyzed. **(C)** MS analysis of the protein has revealed the presence of a unique species, corresponding to the full-length proNGF-YBBR.

As we previously demonstrated (De Nadai et al., 2016), proNGF-YBBR can be produced in *E. coli* and collected from inclusion bodies, similarly to what already reported for the wt, untagged neurotrophin (Rattenholl et al., 2001; Paoletti et al., 2011). Once extracted in denaturing conditions, the tagged neurotrophin is refolded and further purified by ion-exchange FPLC in order to obtain a pure solution of proNGF-YBBR. As indicated in Figure 1, fractions corresponding to the protein elution peak typically contain ≈20 mg of almost pure protein per liter of culture. Mass spectrometry analysis of the same fractions demonstrates that they indeed correspond to the full-length protein (Figure 1C).

**Figure 2:**
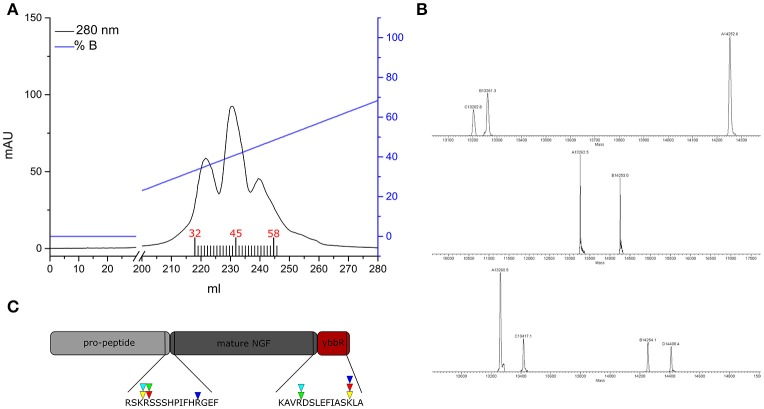
**NGF-YBBR purification and characterization. (A)** Chromatographic profile of FPLC analysis. Black line and blue line represents respectively the absorbance at 280 nm and the percentage of buffer B on the total flowing buffer. Numbers in red identify the fractions used for the MS analysis. **(B)** MS analysis of fractions 32, 45, and 58 has revealed the presence of respectively 3, 2, and 4 different digestion products. **(C)** Schematic illustration of the proNGF-YBBR. The amino acid sequence is highlighted and the proteolytic cleavage sites are identified by the triangles. The color code of the triangles that identify the different NGF species are also reported in Table 1.

To obtain the mature tagged neurotrophin, controlled proteolysis of the purified proNGF-YBBR is performed with trypsin protease. Another step of FPLC chromatography is used for the purification of mature NGF-YBBR. As indicated in Figure 2, the chromatographic run of NGF-YBBR reveals the presence of at least three different species having slightly different elution times. These data suggest that different digestion products arise upon trypsin cleavage. This prompted us to analyze by mass spectrometry (MS) aliquots from the three fractions, which allowed identifying the presence of at least 3, 2, and 4 digestion products, respectively in the first, second and third elution peaks. These correspond to 5 different molecular species (some populate more than one peak), which are fully listed in Table 1; all detected trypsin cleavage sites are highlighted in Figure 2C.

**Table 1:** **The table illustrates the various trypsin digestion products**.

**Name of the specie**	**MW (Da)**	**pI**	**Cleavage site**	**Description of the digestion product**	**Tag**
desoctaNGF−YBBR-2 C-Term	13202.8	8.27	R +9; L +128	Without the first 9 aa at the N-terminus and 2 aa at the C-terminus	Yes
NGF−YBBR -2 C-Term	14252.6	8.56	R −1; L +128	Without 2 aa at the C-terminus	Yes
NGF−Tag YBBR	13261.3	8.81	R −1; R +118	Without 11 aa at the C-terminus	No
NGF−YBBR +R N-Term; -2 C-Term	14408.4	8.8	K −2; L +128	Plus the arginine at the N-terminus and without 2 aa at the C-terminus	Yes
NGF+R N-Term -Tag YBBR	13417.1	9.0	K −2; R +118	Plus the arginine at the N-terminus and without 11 aa at the C-terminus	No

*The first reported specie, lacking the first 9 amino acids, is here termed “DesoctaNGF” because it resembles the naturally occurring desocta form reported for murine NGF (Fahnestock, 1991). The other species are characterized by the lack of two amino acids or of the entire YBBR tag at the C-terminus (-2 C-Term or -Tag YBBR). The species that contains the arginine at the N-Terminus are also shown (+R N-Term). The second and third columns show the molecular weights (Dalton) and the theoretical isoelectric point of the digested species. The amino acids numeration adopted is in accordance to the literature (Paoletti et al., 2009)*.

### fluoproNGF and fluoNGF production

**Figure 3:**
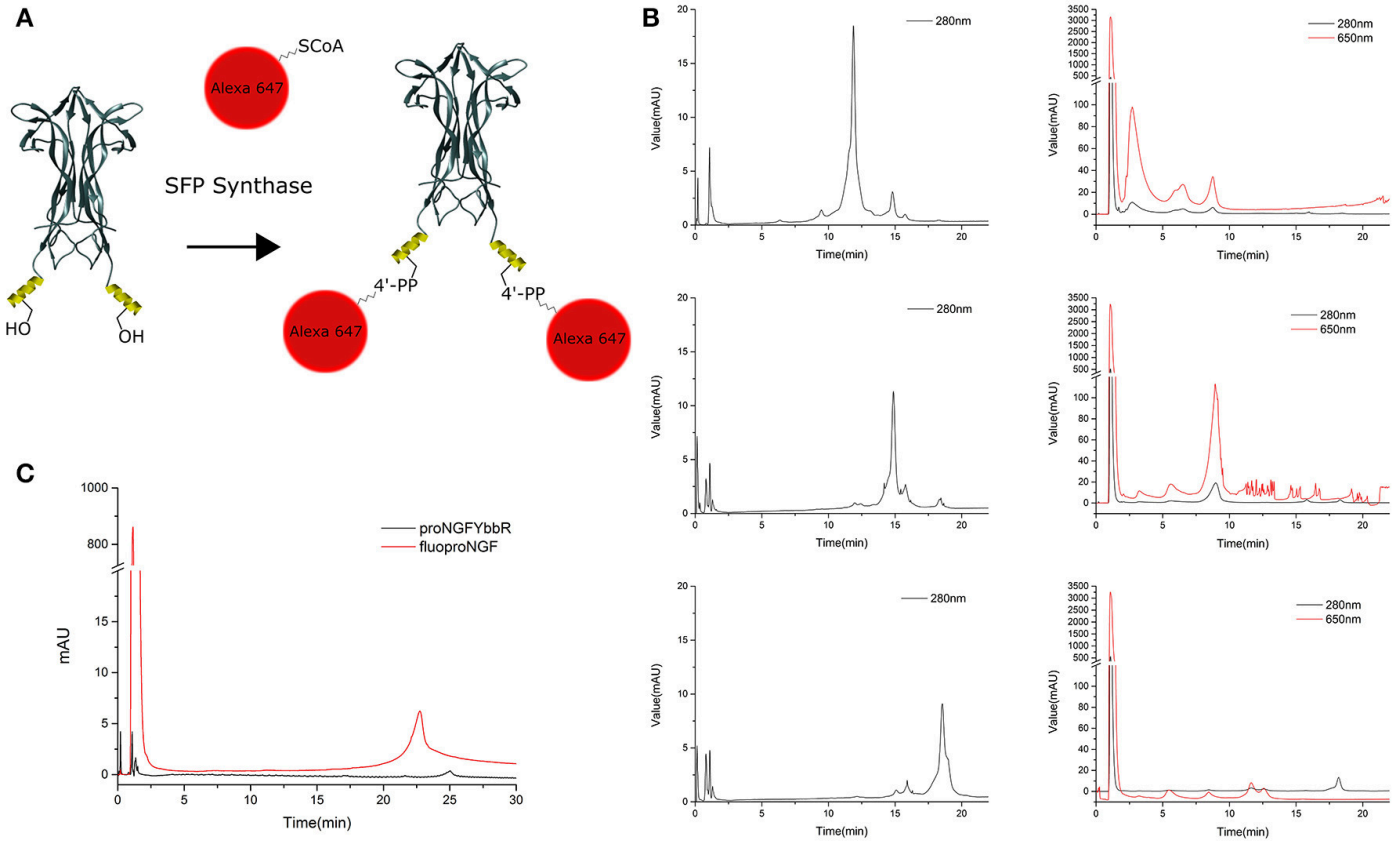
**Labeling strategy and fluoNTs HPLC purification. (A)** Schematic representation of the labeling strategy. Structure of NGF dimer is shown (figure taken from PDB: 1BTG and adapted using UCSF Chimera). The YBBR tag is schematized as an alpha helix and depicted in yellow, side chain of the serine involved in the labeling reaction is also shown. **(B)** Chromatographic profile of the first (top), second (middle), and third (bottom) NGF-YBBR fractions. 18.75 μg of tagged NT were run without performing the labeling reaction. The black line represents the absorbance at 280 nm. The chromatographic profiles of the first, second and third NGF-YBBR fractions after the labeling reaction are also shown, on the right of the respective non-labeled counterparts. Also in this case, 18.75 μg of tagged NT were used for the labeling reaction. Black and red lines represent the absorbance at 280 nm and 650 nm, respectively. **(C)** Superimposed HPLC chromatographic profiles of fluoproNGF and proNGF-YBBR. In all chromatograms, the first peak with about 1 min of retention time is caused by unreacted free fluorophore. The unreacted proNGF-YBBR or NGF-YBBR are virtually undetectable.

Once the proNGF-YBBR and NGF-YBBR are purified, these can be used in a labeling reaction to covalently conjugate *in vitro* each neurotrophin monomer to a single Alexa647 fluorophore (Figure 3A). After the labeling reaction, the fluorescent precursor and mature neurotrophins are processed by ion exchange HPLC to separate the labeled neurotrophins from both the non-reacted ones and the free fluorophores. As for proNGF, the fluorolabeled species can be readily identified (Figure 3C), exploiting the fact that the negative charge of the Alexa647 produces a shift toward a lower retention time in the column. As for NGF-YBBR, optimization of the labeling reaction was required, given that the presence of different but similar digestion products (Figure 2) could lead to a mixture of fluorolabeled functional proteins with undesired not-fluorolabeled species or with poorly-functional ones (Table 1). As the MS analysis performed does not give robust information about the relative abundance of each digestion species, in order to have a better understanding of the protein composition of each of the elution peaks of NGF-YBBR purification, we first run individually the three of them through an ion-exchange HPLC column. Figure 3 outlines the presence of one main species for each fraction. These species are likely different in the three cases as they have a retention time of about 11.8, 14.8, and 18.5 min, respectively. The three fractions were then subjected to the fluorolabeling reaction. We observed a shift of the retention time in column (i.e., the appearance of a fluorolabeled specie) for the first (2.7 min, Figure 3B, upper right graph) and the second (8.9 min, Figure 3B, middle right graph), but not for the third peak (Figure 3B, lower right graph). According to the MS analysis and to the relative predicted isoelectric points, the main species of the first two peaks correspond to desoctaNGF-YBBR -2 C-Term and NGF-YBBR -2 C-Term. Instead, the third fraction likely represents NGF +R N-Term -Tag YBBR as the main species. Given the lack of the YBBR tag, this specie preserves an unaltered retention time after the labeling reaction. Overall, these experiments demonstrated that the second peak of NGF-YBBR FPLC purification was the most suited to be subsequently fluorolabeled. In any case, the additional post-labeling HPLC run is recommended to separate the labeled functional protein from the other resulting from the whole process, which create smaller but non-negligible peaks in the chromatogram as shown in the middle-right panel of Figure 2B.

This protocol allowed us to obtain ~6.3 μg of fluoproNGF and 21.1 μg of fluoNGF, starting respectively from 85 to 90 μg of proNGF-YBBR and NGF-YBBR, a yield of 7.4 and 23.4% respectively. A summary of the whole purification process, with the different yields obtained at every separate step, is reported in Table 2.

**Table 2:** **fluoproNGF and fluoNGF purification yields**.

**Neurotrophin**	**Step 1: FPLC purification to obtain NT-YBBR**			**Step 2: HPLC purification to obtain fluoNT**
proNGF	18 mg per liter of *E. coli* colture (4 mg are stored)			From 85 μg: 6.3 μg
NGF	~3.5 mg per 14 mg of proNGF-YBBR used in the tryptic digestion			From 90 μg: 21.1 μg
	1° elution peak	2° elution peak	3° elution peak	
	1 mg	1.5 mg	0.8 mg	

*Yields obtained at each step of the whole process are reported. In particular, the E. coli expression system typically provides 18 mg of proNGF-YBBR per colture liter after the first purification step. Of these, 4 mg can be stored and 14 mg are used for the production of NGF-YBBR after the trypsin digestion. 3.3 mg of NGF-YBBR are typically obtained, resulting as the combination of 5 different digestion products eluted at three different peaks (see Table 1). In the second step, aliquots of 85 or 90 μg were used to perform the fluoroabeling reaction. After the final HPLC purification, 6.3 or 21.1 μg of pure fluoproNGF or fluoNGF, respectively, can be obtained*.

### Endosomal localization analysis of fluoproNGF and fluoNGF

As a proof of the quality of our fluorolabeled preparations, fluoproNGF and fluoNGF were individually administered within a 3 h interval to differentiated PC12 cells, aiming at characterizing their relative association with endosomal partners involved in their internalization. In detail, we carried out a colocalization analysis of both NGF and proNGF with Rab11, Rab7, Rab5, and EEA1. These proteins have been previously reported to crucially regulate the maintenance/quenching and balance of trophic support during neurotrophin signaling (Saxena et al., 2005; Liu et al., 2007).

**Figure 4:**
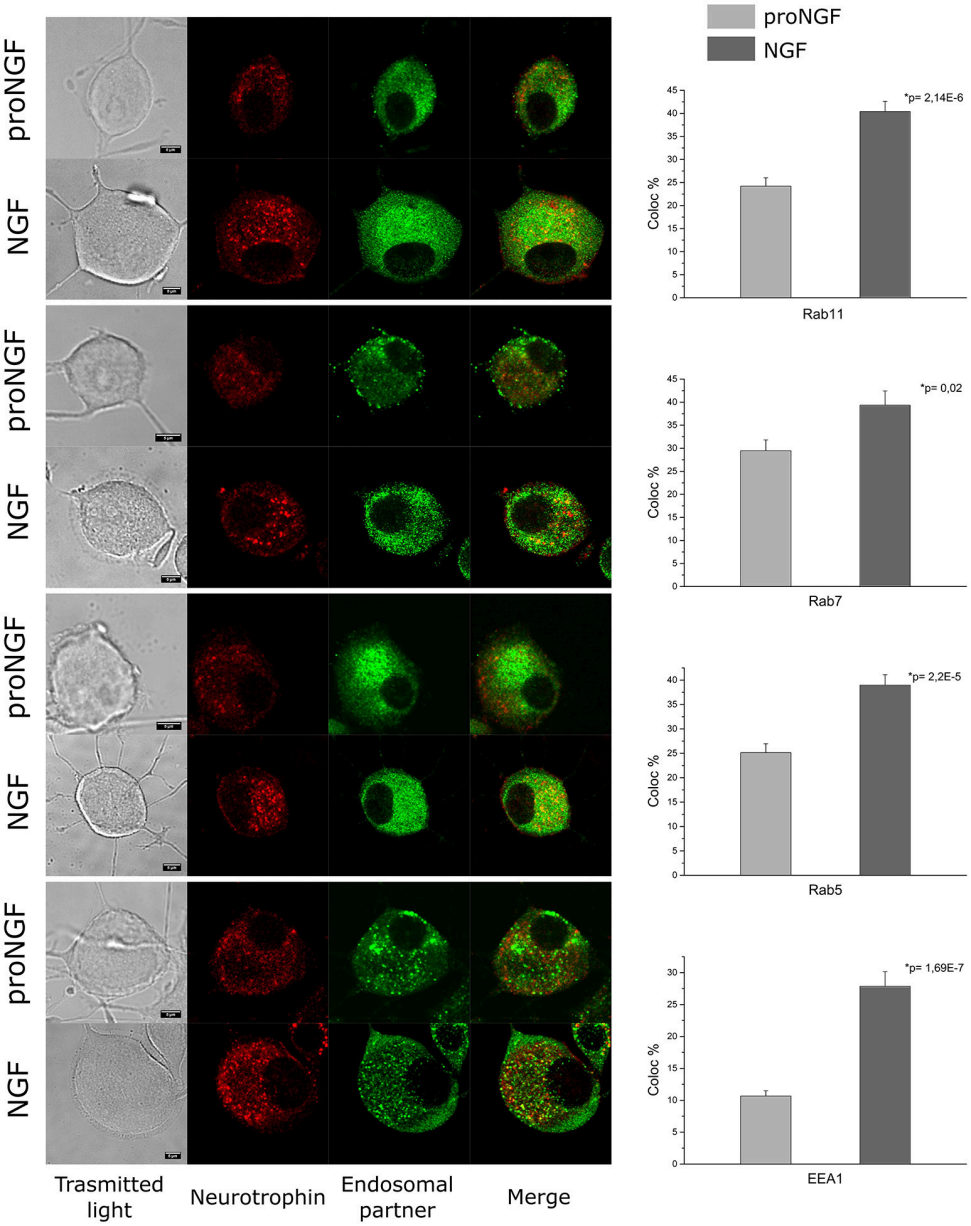
**Colocalization analysis**. PC12 cells imaged by confocal microscopy. Immunocytochemistry experiment showing the colocalization of fluoproNGF or fluoNGF with the endosomal protein partners. Scale bar is 5 μm. On the right of each panel the quantitative analysis of the colocalization of fluoproNGF or fluoNGF with endosomal partners Rab11, Rab7, Rab5, and EEA1 is shown. The histograms show the average colocalization percentage. The color code used is light gray for proNGF and dark gray for NGF. The error bars represent the standard error of the mean and p is the *p*-value.

As shown in Figure 4, first of all we found that endosomes containing NGF have a higher intensity of fluorescence, compared to proNGF ones. This fact is in agreement with our previous observations that a different average number of NTs dimers is contained per endosome in the two cases (De Nadai et al., 2016). Furthermore, we found that proNGF endosomes are almost never detectable in neurites of PC12 cells, contrary to what seen for NGF ones (Supplementary Figure 1). These observations prompted us to develop a colocalization algorithm independent from absolute fluorescence intensity (see Section Materials and Methods) and to restrict our analysis to PC12 soma regions. Our data revealed that the average percentage of colocalization calculated for proNGF with all four endosomal partner considered is significantly different from that observed for NGF. This result will be discussed in light of the opposite biological functions displayed by precursor NGF with respect to the mature form.

## Discussion

The lack of a standardized protocol for the production and purification of fluorolabeled neurotrophins has led to the development of a multitude of different strategies for this purpose (Marchetti et al., 2015; De Nadai et al., 2016). The chemical conjugation of organic dyes to NTs amino- or carboxy- residues (Rosenberg et al., 1986) is maybe the most straightforward of these approaches, but suffers of poor reproducibility. NT chemical biotinylation and further conjugation to Qdot is so far the most used approach (Cui et al., 2007; Wong et al., 2015). However, the vast majority of these approaches does not allow a controlled stoichiometry of labeling, leading to the production of heterogeneously labeled species. Moreover, despite their excellent optical properties, Qdot particles generally have a cumbersome size usually no less than 10 nm in diameter. These aspects are particularly relevant in the neurotrophin-signaling system, which involves the axonal transport of small endosomes containing a limited number of NT dimers, so that even small changes in the number of carried NTs could reasonably lead to drastic alterations in the downstream signaling (De Nadai et al., 2016). Additionally, the presence of a big cargo such as Qdot or the non-homogeneous NTs population could hinder the correct analysis of the intracellular trafficking properties of these proteins, or prevent the study of the dynamics of binding with the cognate receptors.

Our labeling strategy overcomes many of the above-mentioned limitations. In terms of absolute yield, the insertion of the YBBR tag sequence does not affect the protein expression compared to the wt neurotrophin. In fact, 15–20 mg of proNGF-YBBR can be typically obtained per liter of bacterial culture, and this quantity is totally equivalent to the wt, untagged proNGF average yield. Importantly, this yield is also about tenfold higher than typical yields obtained for mono-biotinylatable proNGF-AVItag construct expressed in eukaryotic cells and purified from the cell medium (Sung et al., 2011), which is of 1–1.2 mg/L.

In this paper we characterized the heterogeneous population constituting the mature NGF-YBBR protein used for labeling and designed a procedure to get only the correct fluorolabeled specie. Surprisingly, we found that although the correct NGF species obtained lacks the last two aminoacids of the YBBR tag, this can be still efficiently fluorolabeled by the Sfp enzyme; thus, this will allow the use of a shorter and thus less invasive YBBR tag for future studies. The use of negatively charged fluorophores like Alexa647 in the labeling reaction and the subsequent ion-exchange HPLC purification allow obtaining a pure and homogeneous fluoproNGF and fluoNGF protein population, which is fundamental in order to have a precise control on the cellular response. The optimized method ensures therefore a greater homogeneity and a higher specific activity of the labeled NGF species.

Given the high absolute yield and the great purity grade obtained, this strategy is well suited for several experimental investigations, spanning from single molecule imaging analysis on compartmentalized neuronal cultures (De Nadai et al., 2016), to applications that require large amounts of homogeneous neurotrophin preparations.

Our novel tool was here used to perform a quantitative analysis of the colocalization of the two NGF forms with different endosomal protein partners. It is known that neurotrophins travel inside neurons in signaling endosomes characterized by the presence of different Rab proteins (Matusica and Coulson, 2014). The switching of these endosomal proteins is implicated in the maintenance/quenching of neurotrophins signaling (Saxena et al., 2005; Deinhardt et al., 2006; Liu et al., 2007). Given the opposite biological functions displayed by the mature and precursor forms of NGF, it seems reasonable to hypothesize that these may originate by sub-populations of signaling endosomes having distinct intracellular distributions and sorting, finally leading to stronger (NGF) or weaker (proNGF) trophic actions. Such a finding might be relevant, e.g., for mechanisms linking proNGF/NGF unbalance and neurodegenerative processes relevant for Alzheimer's disease (Tiveron et al., 2013).

The observation that proNGF endosomes are exclusively found in the PC12 soma, contrary to NGF endosomes that are also found in neurites, confirms that the cell biology of the two neurotrophin forms is very different, as previously observed in DRG neurons (De Nadai et al., 2016). Interestingly however, in DRG neurons the proNGF is internalized at the axon tips (from where it is transported to the soma), while it is internalized at the soma level but not anterogradely transported. It remains to be seen whether the transport properties of proNGF and NGF are also different in PC12 cells. In any case, the different internalization pattern in PC12 and DRG neurons might be ascribed to the fact that PC12 neurite processes are not differentiated in axons or dendrites and thus might be functionally different from the fully polarized processes of DRG neurons.

The analysis of the colocalization between the two fluorescent-NTs and the endosomal protein partners did not highlight a preference in the association of proNGF with any of the protein partners considered. The average colocalization percentage of proNGF was found to be about half of that seen for NGF for all protein partners investigated. Although, many other Rab family members were not considered in this work, our data suggest that proNGF could undergo a different intracellular trafficking compared to that of NGF. In particular, the anti-Rab5 antibody used in this work recognizes specifically one isoform (A) out of three (A, B, C). These endosomal proteins are considered master regulators of the formation and sorting of early endosome; it was observed that Rab5A and Rab5B are directly involved in the trafficking of the epidermal growth factor receptor (a prototype tyrosine kinase receptor), while Rab5C have a compensatory function (Chen et al., 2009). Therefore, we cannot exclude that proNGF could associate with other Rab5 isoforms involved in compensatory functions with respect to those afforded by Rab5A/NGF positive endosomes.

Further work will be necessary for the complete characterization of the molecular composition of the *signaling endosomes* populated by proNGF or NGF. The versatility of the labeling strategy presented in this work could represent a powerful tool to achieve this goal. For example, each neurotrophin could be in principle also biotinylated and further conjugated to streptavidin-coated magnetic nanoparticles, in order to purify and analyze the respective *signaling endosomes*.

## Author contributions

PD, LM, and AC designed the study; PD and MC performed the experiments; all authors analyzed the data; PD, SL, LM, and AC wrote the manuscript.

## Funding

Grants from Alzheimer Drug Discovery Foundation (ADDF, grant#20120601) and EU PAINCAGE FP7 Collaborative Project num. 603191 to AC are gratefully acknowledged.

### Conflict of interest statement

The authors declare that the research was conducted in the absence of any commercial or financial relationships that could be construed as a potential conflict of interest.
