# Supplementary material for: An Optimized Procedure for the Site-Directed Labeling of NGF and proNGF for Imaging Purposes
Source: Front Mol Biosci. 2017 Feb 2;4:4. doi: 10.3389/fmolb.2017.00004 (PMC5288393; doi:10.3389/fmolb.2017.00004)
Supplement: Supplementary file 1 [file Image1.PDF]

*Supplementary Material*

# An Optimized Procedure for the Site-directed Labelling of NGF and proNGF for Imaging Purposes

Pierluigi Di Matteo[1], Mariantonietta Calvello[1], Stefano Luin[2], Laura Marchetti[1,3], Antonino Cattaneo[1,*]

[1]BioSNS Laboratory, Scuola Normale Superiore and Istituto di Neuroscienze – CNR, Pisa, Italy

[2] NEST Laboratory, Scuola Normale Superiore and Istituto Nanoscienze – CNR Pisa, Italy

[3] Center for Nanotechnology Innovation@NEST, Istituto Italiano di Tecnologia, Pisa, Italy

*** Correspondence:** Antonino Cattaneo: antonino.cattaneo@sns.it

## 1 Supplementary Figures

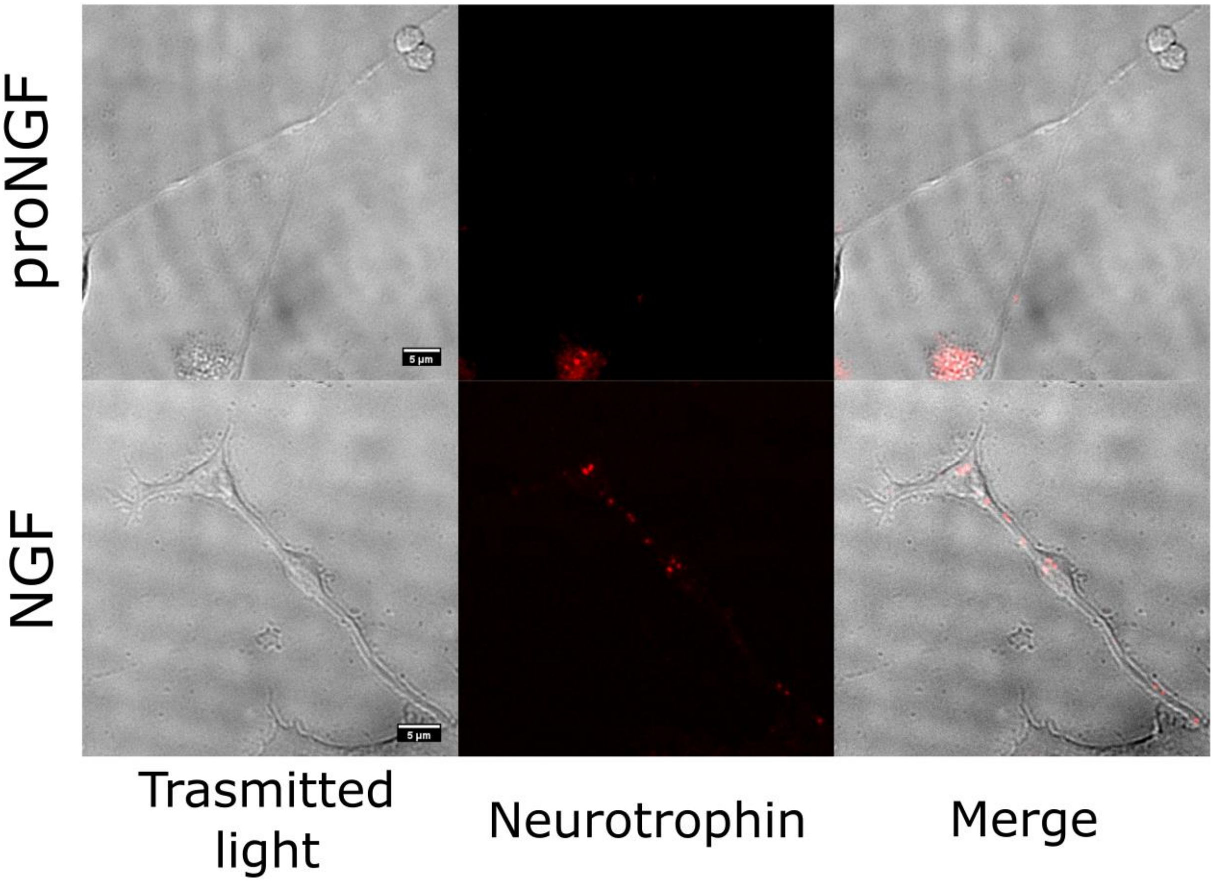


**Supplementary Figure 1.** Neurotrophins vesicles in neurites. Images of differentiated PC12 cells captured by laser scanning confocal microscopy showing the absence of vesicles containing fluorescent neurotrophins upon fluoproNGF administration, upper panels. Images also show the presence of several vesicles containing fluoNGF inside neurites, lower panels. Scale bar is 5 µm.
